# Thermoelectric transports in pristine and functionalized boron phosphide monolayers

**DOI:** 10.1038/s41598-021-89579-5

**Published:** 2021-05-11

**Authors:** Min-Shan Li, Dong-Chuan Mo, Shu-Shen Lyu

**Affiliations:** 1 School of Chemical Engineering and Technology, Sun Yat-Sen University, Guangzhou, 510275 People’s Republic of China; 2 School of Materials, Sun Yat-Sen University, Guangzhou, 510275 People’s Republic of China; 3 Guangdong Engineering Technology Research Centre for Advanced Thermal Control Material and System Integration (ATCMSI), Sun Yat-Sen University, Guangzhou, 510275 People’s Republic of China

**Keywords:** Two-dimensional materials, Thermoelectrics

## Abstract

Recently, a new monolayer Group III–V material, two-dimensional boron phosphide (BP), has shown great potential for energy storage and energy conversion applications. We study the thermoelectric properties of BP monolayer as well as the effect of functionalization by first-principles calculation and Boltzmann transport theory. Combined with a moderate bandgap of 0.90 eV and ultra-high carrier mobility, a large *ZT* value of 0.255 at 300 K is predicted for two-dimensional BP. While the drastically reduced thermal conductivity in hydrogenated and fluorinated BP is favored for thermoelectric conversion, the decreased carrier mobility has limited the improvement of thermoelectric figure of merit.

## Introduction

Thermoelectric material, which can directly convert waste heat into electricity, provides a promising solution for global issues like energy crisis^1–4^. The thermoelectric performance of a material can be characterized by a dimensionless figure of merit:

1 $$ZT=\frac{{S}^{2}\sigma T}{{k}_{\text{el}}+{k}_{\text{la}}},$$

where *S* is the Seebeck coefficient, *σ* is the electronic conductivity, *k*_el_ and *k*_la_ are the thermal conductivities contributed by electrons and phonons, respectively. To obtain high thermoelectric performance, a material generally needs to have high electrical conductivity *σ*, high Seebeck coefficient *S* and low thermal conductivity *k* at the same time^5^. While the inter-coupling of the electronic parameters has made the optimization of thermoelectric performance a great challenge, early studies suggest that lowering the dimension of materials appears to be an effective approach^6–9^. Over the past few decades, the discovery of graphene has driven the exploration, fabrication and measurement techniques of two-dimensional materials^10–17^. In order to extract the thermal conductivity in two-dimensional materials, extensive efforts^18,19^ have been devoted to the development of 3*ω* method, Raman spectroscopy method and so on. Qiu et al.^20^ proposed a modified 3*ω* method for the measurement of thermal conductivity in non-conductive fiber. Balandin et al.^21^ conducted the measurement of suspended graphene monolayer using Raman spectroscopy method. As for thermoelectric performance, Fei et al.^22^ reported the orthogonal electronic and thermal transport in black phosphorene and a high *ZT* value of above 1 at 300 K. The room temperature thermoelectric figure of merit for other two-dimensional materials, such as transition metal dichalcogenides (0.7–0.9)^23^, Tellurium (0.54–0.8)^24,25^ and PdSe_2_ (1.1)^26^, have also been studied. However, many of the currently developed two-dimensional thermoelectric materials either suffer from low performance or are based on toxic or rare elements. Given the increasing demand for portable and wearable thermoelectric devices^27,28^, the search for eco-friendly and elementally abundant high-performance thermoelectric materials has become an increasingly pressing issue.

Recently, a new monolayer Group III–V material, two-dimensional boron phosphide (BP), with good thermodynamic stability, wide bandgap and ultra-high carrier mobility^29,30^, has shown great potential for energy storage and energy conversion applications. The experimental realization of crystalline BP thin film has further suggested its possibility in practical use. Unfortunately, the thermal conductivity of 220–323 W/mK in BP monolayer^31,32^ is rather high compared with other two-dimensional thermoelectric materials. To address this problem, the idea of using van der Waals interaction to reduce the lattice thermal conductivity has been applied. Mohanta et al.^33^ designed a bilayer BP/MoS_2_ heterojunction and reported a *ZT* value of up to 1.1 for *p*-type doping at 300 K. A theoretical *ZT* value as high as 1.8 at 1200 K in bilayer BP is also reported^32^. Apart from constructing van der Waals heterojunction, surface functionalization is another effective method to modulate the thermal and electronic transport properties in low-dimensional materials. For planar two-dimensional materials, the contribution of ZA phonons to the thermal transport can be reduced by functionalization due to the broken reflection symmetry, and thus leading to a reduced thermal conductivity^34,35^. Recent theoretical study has also reported the enhancement of electronic figure of merit *ZT*_e_ in silicene by hydrogenation^36^. Yet, little research has been done on the thermoelectric properties of two-dimensional BP, and the effect of functionalization has also been little discussed.

Herein, the thermoelectric transport properties of two-dimensional BP as well as the effect of functionalization are investigated systematically by a first-principles approach in combination with Boltzmann transport theory. Our study provides insights into the thermoelectric properties of BP monolayer and the effect of functionalization.

## Results and discussions

**Figure 1:**
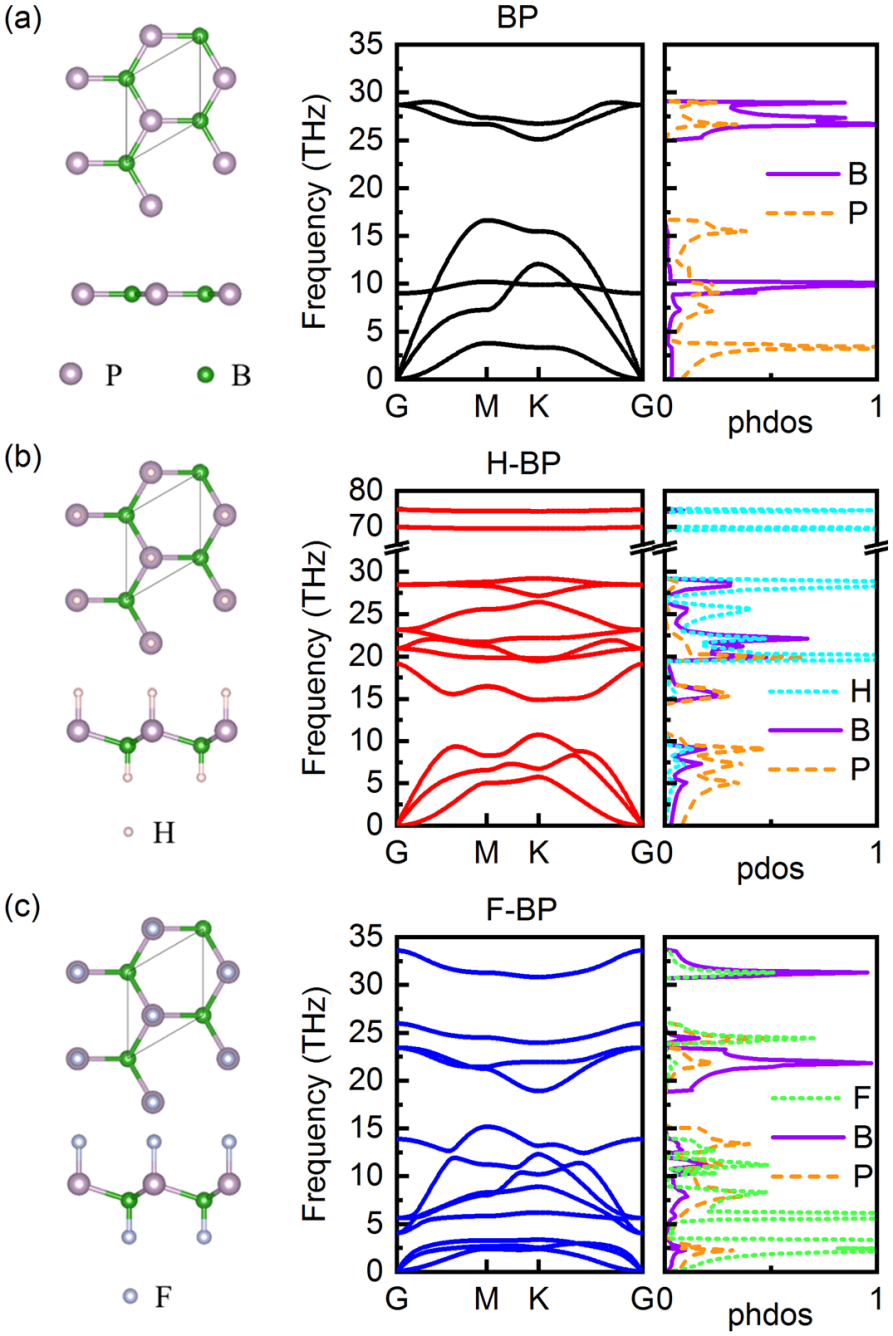
Top view and side view for (**a**) BP, (**b**) H-BP and (**c**) F-BP with corresponding phonon dispersions and density of states.

The optimized structures of BP and functionalized BP are shown in Fig. 1. Similar to h-BN, two-dimensional BP has a planar honeycomb structure with one boron atom and one phosphorus atom in a unit cell. The obtained lattice constant for BP is 3.21 Å. After functionalization, the planar structure is distorted. H-BP and F-BP exhibit slightly larger lattice constants (3.23 Å and 3.29 Å) and a low-buckled configuration. The B–P sublayer buckling height is 0.62 Å for F-BP and 0.56 Å for H-BP. These results are in good agreement with previous work^37^. The calculated in-plane stiffness *C*_2D_ for BP, H-BP and F-BP are 137.31 J/m^2^, 105.72 J/m^2^ and 89.39 J/m^2^, respectively. The *C*_2D_ of the modified material decreases due to the presence of the buckled structure, indicating that the material is easier to deform under tension. In particular, F-BP has greater buckling and a smaller *C*_2D_.

The phonon dispersions and density of states are also shown in Fig. 1. For BP, the acoustic branches have a wide range from 0 to 17 THz and are coupled with the out-of-plane flexure optical (ZO) modes, which will result in strong acoustic-optical phonon scattering. After functionalization, the acoustic branches are compressed to below 11 THz in H-BP and below 3.5 THz in F-BP. Besides, the ZO mode in H-BP and F-BP is no longer coupled with the acoustic branches due to the buckling effect^38^. The presence of an acoustic-optical gap is beneficial to suppress the optical-acoustic phonon scattering. As observed in the phonon density of states, the light H atom contributes a lot in high-frequency vibrations while the heavy F atom affects the low-frequency phonon modes greatly.

**Figure 2:**
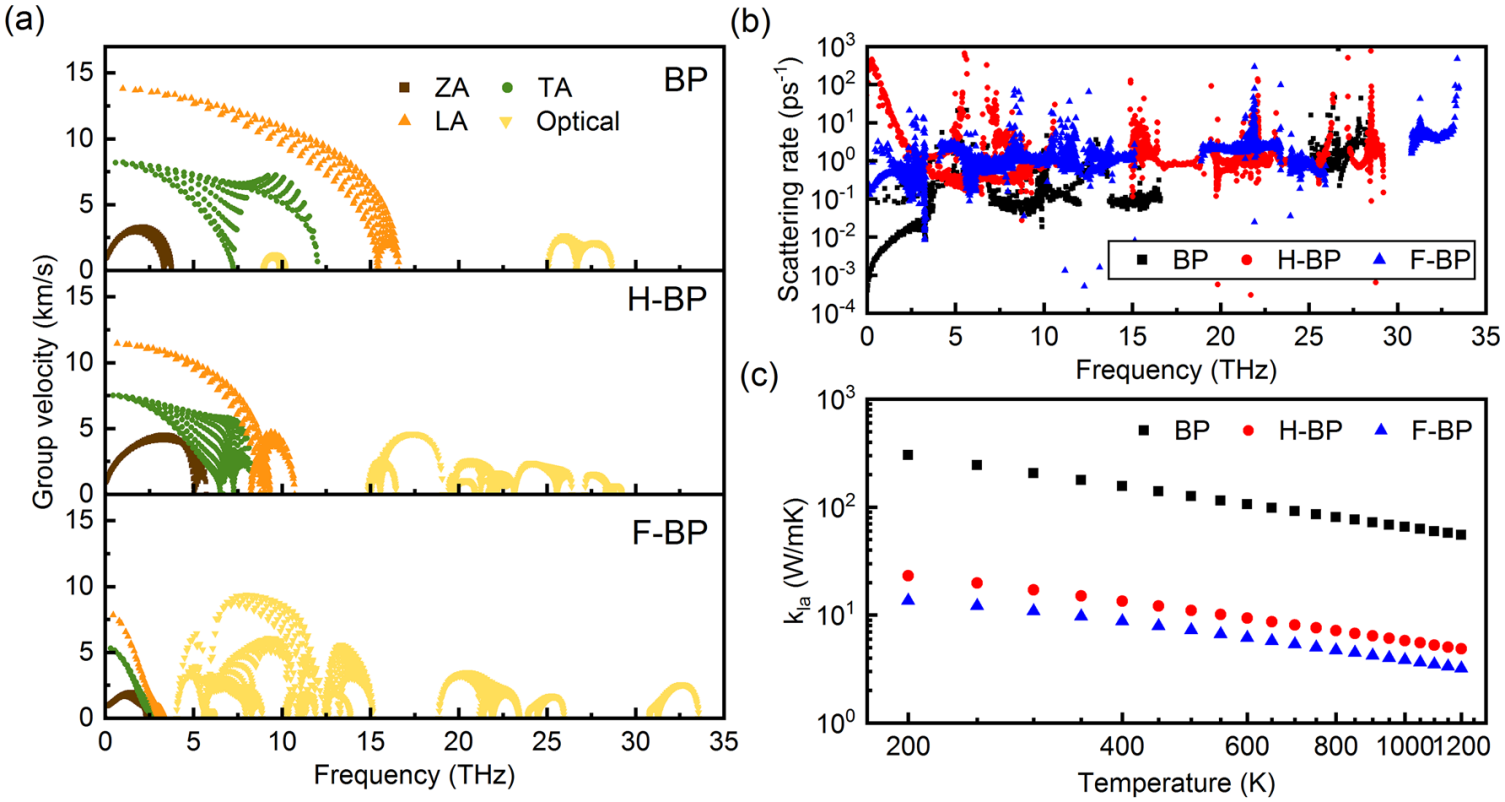
(**a**) Phonon group velocities *v*_g_ and (**b**) scattering rates as a function of frequency; and (**c**) lattice thermal conductivity *k*_la_ as a function of temperature for BP, H-BP and F-BP.

It is noted that low-frequency phonons have a major effect on the thermal transport and thus we focus on the acoustic phonons. As illustrated in Fig. 2a, the phonon group velocities *v*_g_ of acoustic phonons in H-BP are close to those in BP, while those in F-BP shows a substantial decrease. Such a decrease is owing to the severe condense of acoustic vibrations and will reduce the *k*_la_. The phonon scattering rates, as shown in Fig. 2b, are dramatically increased in low-frequency region after functionalization, especially for H-BP. The phonon scattering rate is determined by the scattering intensity and the scattering possibility, where the former can be characterized by the Grüneisen parameter and the latter by the phase space. Although BP has a larger phase space (see Supplementary Fig. S1 online), the enhanced anharmonicity of ZA phonons in functionalized BP leads to the increased scattering rates in the low-frequency region, especially for H-BP.

Figure 2c summarizes the lattice thermal conductivity *k*_la_ with respect to temperature. At 300 K, the calculated *k*_la_ of BP monolayer is 205.3 W/mK. Unlike the common trend where two-dimensional materials possess higher *k*_la_ than their bulk compounds^21^, the *k*_la_ of BP monolayer is lower than the measured result for BP crystal (460–490 W/mK)^39,40^, which may due to the different atomic arrangements in monolayer BP and bulk BP. Similar to graphene, the ZA phonons dominate the thermal transport among all the phonons in BP. The contribution of the ZA mode to the thermal conductivity is up to 51.80%. After functionalization, the *k*_la_ is reduced by one order of magnitude. The calculated *k*_la_ at 300 K is 17.1 W/mK and 10.8 W/mK for H-BP and F-BP, respectively. The buckling structure has broken the out-of-plane symmetry and increased the scattering rates of the ZA phonons, and thus the contribution of ZA phonons is reduced to 7.20% and 7.30% in H-BP and F-BP, respectively. It is noted that different primary structures and relaxation algorithms may lead to a difference in the distorted functionalized structures after relaxation, and thus the scattering situation of ZA phonons could be different. Since the contribution of ZA phonons to thermal conductivity is dominant in two-dimensional planar materials, the thermal conductivity could be affected.

**Figure 3:**
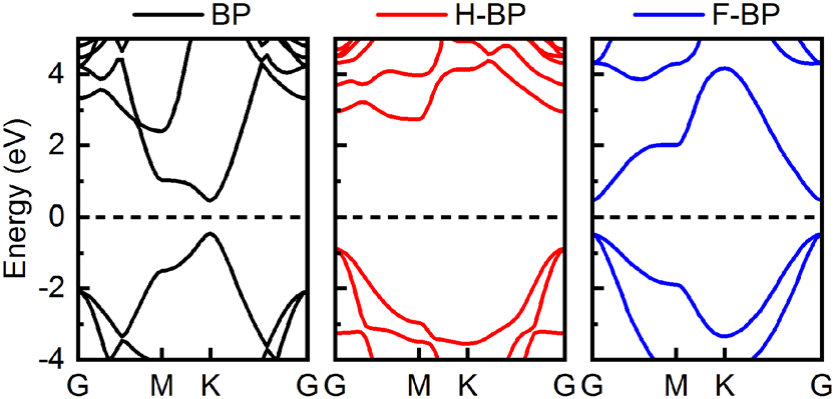
Electronic band structure for BP, H-BP and F-BP.

To investigate the electronic properties, the band structures are computed and plotted in Fig. 3. BP has a direct-bandgap of 0.90 eV with both the conduction band minimum (CBM) and valence band maximum (VBM) located at the K point. According to the projected density of states (see Supplementary Fig. S2 online), the CBM (VBM) is mainly contributed by the pz orbital of boron (phosphorus) atom and the p-orbital of boron and phosphorus atom hybridize strongly with each other. After functionalization, H-BP has an indirect bandgap of 3.63 eV and F-BP has a direct bandgap of 0.94 eV. The wide bandgaps in these materials are beneficial in thermoelectric application since the bipolar electronic thermal conductivity can be suppressed. The flat band presented around the CBM in H-BP as well as the degenerated VBM in H-BP and F-BP can lead to a large density of states, and thus a large effective mass. We obtained the effective electron (hole) masses at CBM (VBM) along the high-symmetry path direction by fitting the quadratic function (see Supplementary Table S1). The enhancement in effective masses for functionalization BP is favorable for obtaining a high Seebeck coefficient but it may reduce the carrier mobility.

**Figure 4:**
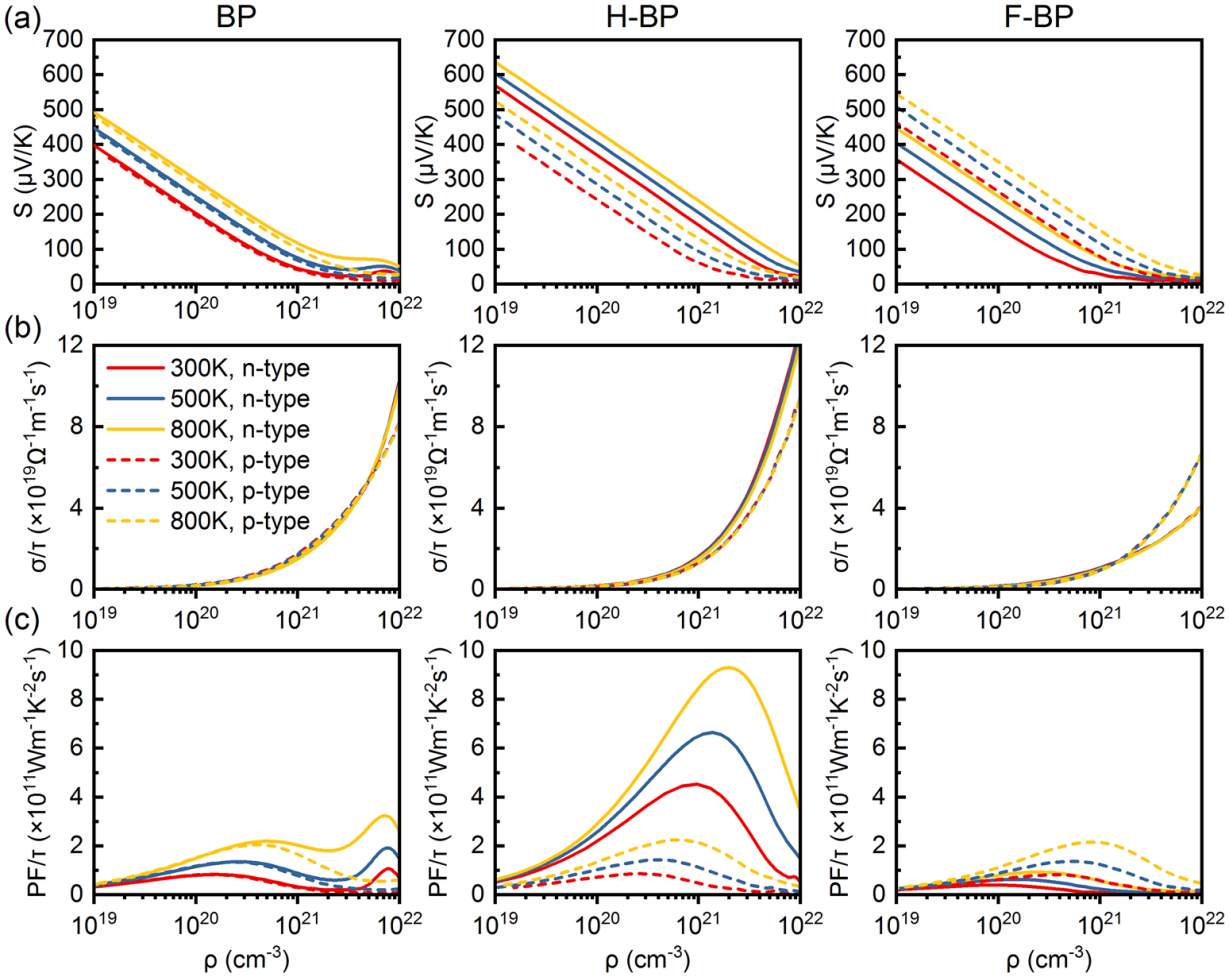
(**a**) The Seebeck coefficient *S*, (**b**) electronic conductivity *σ/τ* and (**c**) power factor *PF/τ* as a function of carrier concentration *ρ* for BP, H-BP and F-BP, respectively. The solid lines represent *n*-type doping and the dash lines represent *p*-type doping.

Figure 4 illustrates the Seebeck coefficient *S*, electronic conductivity *σ/τ* and thermoelectric power factor *PF/τ* as a function of the carrier concentration *ρ* for *n*-type and *p*-type doping. BP exhibits slightly larger *S* for *n*-type doping and a peak value of 1526/μVK at 300 K is obtained. After functionalization, H-BP shows significantly enhanced *S* for both type and F-BP shows better performance for *p*-type doping. While the S increases with increasing temperature, temperature has little effect on the *σ/τ*. F-BP has a smaller *σ/τ*, which may due to the strong electronegativity of F atom. The inter-related parameters, *S* and *σ/τ*, display different trends and magnitude of variation with increasing carrier concentration, making the optimization of *PF*/*τ* challenging. Since the *PF/τ* is proportional to the square of *S*, the increased *S* in H-BP has led to the significant improvement of *PF/τ* for *n*-type doping.

**Figure 5:**
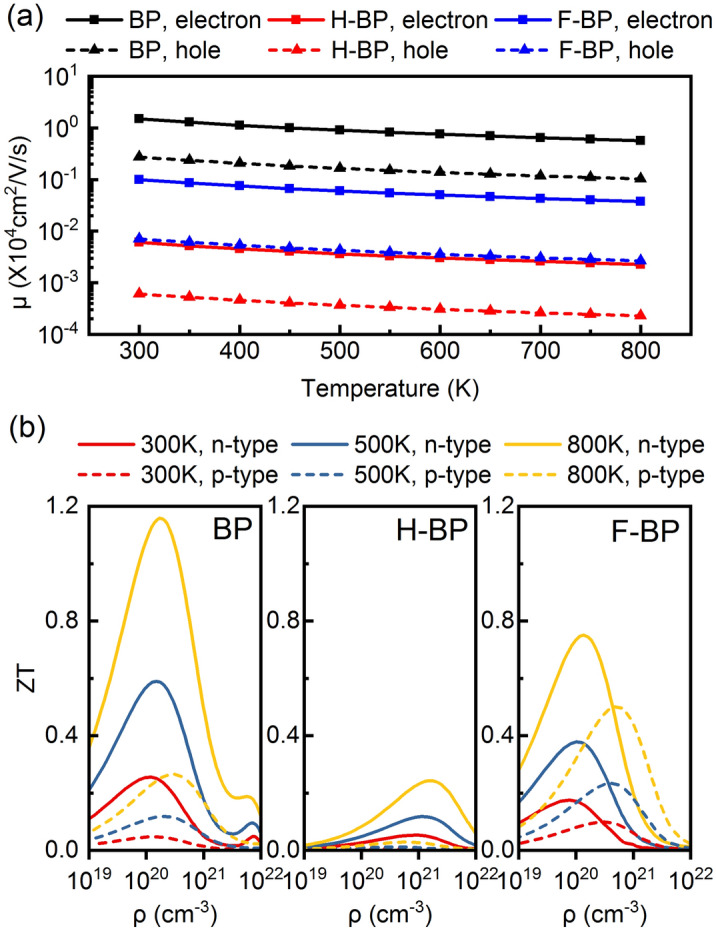
(**a**) The carrier mobility *μ* and (**b**) the figure of merit *ZT* with respect to different temperature as a function of carrier concentration *ρ* for BP, H-BP and F-BP. The solid lines represent *n*-type doping and the dash lines represent *p*-type doping.

To better analyze the thermoelectric performance, we calculated the carrier mobility *μ* through the deformation potential theory^41^:

2 $${\mu }_{2D}=\frac{2e{\hslash }{C}_{2D}}{2{\text{k}}_{\text{B}}T{m}^{*2}{E}_{d}^{2}},$$

where e, ℏ, k_B_, T, m^*^ and E_d_ are the electron charge, reduced Planck constant, Boltzmann constant, temperature, band effective mass and deformation potential constant, respectively. As presented in Fig. 5a, the carrier mobility of BP is 1.50 × 10^4^ cm^2^/V/s for electron and 2.74 × 10^3^ cm^2^/V/s for hole at 300 K. These result are in accord with previous studies reported by Xie et al. (1.37–6.88 × 10^4^ cm^2^/V/s)^30^, Zeng et al. (0.45–1.36 × 10^4^ cm^2^/V/s)^42^ and Mohanta et al. (0.62–5.77 × 10^4^ cm^2^/V/s)^33^. The ultra-high carrier mobility of BP is comparable to other high-mobility materials such as graphene (~ 10^5^ cm^2^/V/s)^43^ and black phosphorene (~ 10^5^ cm^2^/V/s)^44–46^. For H-BP, the enhanced scattering of phonons to electrons and holes, as indicated by the *E*_d_, has led to the drastically reduced carrier mobility. For F-BP, the carrier mobility is also reduced by one order of magnitude. Such decrease is also affected by the crank structure and is not limited or controlled. The electronic relaxation time is further obtained through Eq. (3)

3 $$\mu =e\tau /{m}^{*}$$

The large decrease in carrier mobility leads to a smaller carrier relaxation time in functionalized BP (see Supplementary Table S1).

Figure 5b illustrates the thermoelectric figure of merit under different temperature. The *ZT* values for *n*-type doping are higher than the performance for *p*-type doping. The maximum *ZT* of BP at 300 K is 0.255 for *n*-type doping and 0.046 for *p*-type doping. As temperature rises, the *ZT*_max_ can be further increased, reaching 0.589 (0.119) and 1.16 (0.265) at 500 K and 800 K for *n* (*p*)-type doping. The high *ZT* values make BP monolayer a competitive candidate for thermoelectric applications, especially when compared with other two-dimensional materials containing heavy atoms (α-tellurium, 0.54 at 300 K^25^; antimonene, 0.58 at 300 K^47^). For functionalized BP, the *ZT*_max_ for H-BP and F-BP at 300 K are 0.053 and 0.321, respectively. The thermoelectric performance is reduced after hydrogenation while the *ZT* value for *p*-type doping can be enhanced by fluorination. Since the enhanced scattering of phonons on carriers would significantly reduce the carrier mobilities and carrier relaxation time, surface functionalization may not be an efficient way to improve the thermoelectric performance in planar two-dimensional materials when compared with doping^48^, constructing nanoparticle-aligned structures^49^ and heterojunctions^32,33^.

## Conclusion

In conclusion, the thermoelectric transport properties of two-dimensional BP monolayer as well as the effect of functionalization have been investigated by means of first-principles calculation and the Boltzmann transport theory. Compared with other two-dimensional materials, BP shows a high *ZT* value of up to 0.255 at 300 K, making it a promising candidate. While hydrogenation and fluorination have reduced the thermal conductivities by one order of magnitude, the improvement of thermoelectric properties is limited due to the enhanced scattering of phonons on carriers and significantly reduced carrier relaxation times.

## Methods

The density functional theory calculations are performed using the Vienna Ab-initio Simulation Package (VASP)^50,51^. The generalized gradient approximation (GGA) of Perdew–Burke–Ernzerhof (PBE)^52^ is adopted to describe the exchange–correlation functional. The energy cutoff is set as 500 eV. Using a 19 × 19 × 1 k-mesh, the structures are fully relaxed with an energy and force converge threshold of 10^–8^ eV and 10^–5^ eV/Å, respectively. A vacuum of more than 15 Å along the out-of-plane direction is applied to prevent the interaction between periodic image layers. Based on the density functional perturbation theory (DFPT), the harmonic phonons are calculated using the VASP and Phonopy package^53^ with a 5 × 5 × 1 supercell. For anharmonic phonons, a 3 × 3 × 1 supercell is adopted in the calculation. The phonon thermal conductivity *k*_la_ is obtained through iteratively solving the linearized phonon Boltzmann transportation equation (BTE), as implemented in the ShengBTE code^54^. The electronic transport properties, such as Seebeck coefficient *S*, electronic conductivity *σ* and electronic thermal conductivity *k*_el_, are calculated using the constant relaxation time approximation (CRTA) and linearized BTE, as implemented in the BoltzTraP2 code^55^. A dense *k*-mesh of 251 × 251 × 1 is employed during the calculation. As the layer thickness of two-dimensional material is not well defined, a layer thickness of 3.57 Å (optimized interlayer distance in bilayer BP)^32^ is adopted in our calculations.

## Supplementary Information

Supplementary Information.

## References

[1] Disalvo FJ (1999). Thermoelectric cooling and power generation. Science.

[2] Bell LE (2008). Cooling, heating, generating power, and recovering waste heat with thermoelectric systems. Science.

[3] He J, Tritt TM (2017). Advances in thermoelectric materials research: Looking back and moving forward. Science.

[4] Yang L, Chen ZG, Dargusch MS, Zou J (2018). High performance thermoelectric materials: Progress and their applications. Adv. Energy Mater..

[5] Tan G, Zhao LD, Kanatzidis MG (2016). Rationally designing high-performance bulk thermoelectric materials. Chem. Rev..

[6] Hicks L, Dresselhaus MS (1993). Effect of quantum-well structures on the thermoelectric Bgure of merit. Phys. Rev. B. Condens. Matter.

[7] Hicks L, Dresselhaus MS (1993). Thermoelectric figure of merit of a one-dimensional conductor. Phys. Rev. B. Condens. Matter.

[8] Boukai AI (2008). Silicon nanowires as efficient thermoelectric materials. Nature.

[9] Hochbaum AI (2008). Enhanced thermoelectric performance of rough silicon nanowires. Nature.

[10] Novoselov KS, Geim AK, Morozov SV, Jiang D, Zhang Y, Dubonos SV, Grigorieva IV, Firsov AA (2004). Electric field effect in atomically thin carbon films. Science.

[11] Kim KS (2009). Large-scale pattern growth of graphene films for stretchable transparent electrodes. Nature.

[12] Splendiani A (2010). Emerging photoluminescence in monolayer MoS2. Nano Lett..

[13] Coleman JN (2011). Two-dimensional nanosheets produced by liquid exfoliation of layered materials. Science.

[14] Wang QH, Kalantar-Zadeh K, Kis A, Coleman JN, Strano MS (2012). Electronics and optoelectronics of two-dimensional transition metal dichalcogenides. Nat. Nanotechnol..

[15] Radisavljevic B, Radenovic A, Brivio J, Giacometti V, Kis A (2011). Single-layer MoS2 transistors. Nat. Nanotechnol..

[16] Chhowalla M (2013). The chemistry of two-dimensional layered transition metal dichalcogenide nanosheets. Nat. Chem..

[17] Qiu L, Zhu N, Feng Y, Zhang X, Wang X (2020). Interfacial thermal transport properties of polyurethane/carbon nanotube hybrid composites. Int. J. Heat Mass Transf..

[18] Qiu L (2019). Enhancing the interfacial interaction of carbon nanotubes fibers by Au nanoparticles with improved performance of the electrical and thermal conductivity. Carbon N. Y..

[19] Qiu L (2020). A review of recent advances in thermophysical properties at the nanoscale: From solid state to colloids. Phys. Rep..

[20] Qiu L, Ouyang Y, Feng Y, Zhang X (2018). Note: Thermal conductivity measurement of individual porous polyimide fibers using a modified wire-shape 3 ω method. Rev. Sci. Instrum..

[21] Balandin AA (2008). Superior thermal conductivity of single-layer graphene. Nano Lett..

[22] Fei R (2014). Enhanced thermoelectric efficiency via orthogonal electrical and thermal conductances in phosphorene. Nano Lett..

[23] Chen KX, Wang XM, Mo DC, Lyu SS (2015). Thermoelectric properties of transition metal dichalcogenides: From monolayers to nanotubes. J. Phys. Chem. C.

[24] Sharma S, Singh N, Schwingenschlögl U (2018). Two-dimensional tellurene as excellent thermoelectric material. ACS Appl. Energy Mater..

[25] Gao Z, Liu G, Ren J (2018). High thermoelectric performance in two-dimensional tellurium: An ab initio study. ACS Appl. Mater. Interfaces.

[26] Qin D (2018). Monolayer PdSe2: A promising two-dimensional thermoelectric material. Sci. Rep..

[27] Jin Q (2019). Flexible layer-structured Bi_2_Te_3_ thermoelectric on a carbon nanotube scaffold. Nat. Mater..

[28] Wang Y (2019). Flexible thermoelectric materials and generators: Challenges and innovations. Adv. Mater..

[29] Çakir D, Kecik D, Sahin H, Durgun E, Peeters FM (2015). Realization of a p–n junction in a single layer boron-phosphide. Phys. Chem. Chem. Phys..

[30] Xie M (2016). Two-dimensional BX (X = P, As, Sb) semiconductors with mobilities approaching graphene. Nanoscale.

[31] Fan H, Wu H, Lindsay L, Hu Y (2019). Ab initio investigation of single-layer high thermal conductivity boron compounds. Phys. Rev. B.

[32] Zhou ZZ, Liu HJ, Fan DD, Cao GH, Sheng CY (2019). High thermoelectric performance in the hexagonal bilayer structure consisting of light boron and phosphorus elements. Phys. Rev. B.

[33] Mohanta MK (2020). Interfacing boron monophosphide with molybdenum disulfide for an ultrahigh performance in thermoelectrics, two-dimensional excitonic solar cells, and nanopiezotronics. ACS Appl. Mater. Interfaces.

[34] Lindsay L, Broido DA, Mingo N (2010). Flexural phonons and thermal transport in graphene. Phys. Rev. B Condens. Matter Mater. Phys..

[35] Seol JH (2010). Two-dimensional phonon transport in supported graphene. Science.

[36] Li YF, Tang GH, Fu B (2019). Hydrogenation: An effective strategy to improve the thermoelectric properties of multilayer silicene. Phys. Rev. B.

[37] Ullah S, Denis PA, Sato F (2018). Hydrogenation and fluorination of 2D boron phosphide and boron arsenide: A density functional theory investigation. ACS Omega.

[38] Peng B (2017). The conflicting role of buckled structure in phonon transport of 2D group-IV and group-V materials. Nanoscale.

[39] Zheng Q (2018). High thermal conductivity in isotopically enriched cubic boron phosphide. Adv. Funct. Mater..

[40] Kang JS, Wu H, Hu Y (2017). Thermal properties and phonon spectral characterization of synthetic boron phosphide for high thermal conductivity applications. Nano Lett..

[41] Xi J, Long M, Tang L, Wang D, Shuai Z (2012). First-principles prediction of charge mobility in carbon and organic nanomaterials. Nanoscale.

[42] Zeng B (2016). First-principles prediction of the electronic structure and carrier mobility in hexagonal boron phosphide sheet and nanoribbons. J. Phys. Chem. C.

[43] Morozov SV (2008). Giant intrinsic carrier mobilities in graphene and its bilayer. Phys. Rev. Lett..

[44] Liu H (2014). Phosphorene: An unexplored 2D semiconductor with a high hole mobility. ACS Nano.

[45] Li L (2014). Black phosphorus field-effect transistors. Nat. Nanotechnol..

[46] Qiao J, Kong X, Hu ZX, Yang F, Ji W (2014). High-mobility transport anisotropy and linear dichroism in few-layer black phosphorus. Nat. Commun..

[47] Sharma S, Kumar S, Schwingenschlögl U (2017). Arsenene and antimonene: two-dimensional materials with high thermoelectric figures of merit. Phys. Rev. Appl..

[48] Chandra S, Banik A, Biswas K (2018). N-Type ultrathin few-layer nanosheets of Bi doped SnSe: Synthesis and thermoelectric properties. ACS Energy Lett..

[49] Qiu L (2018). Iodine nanoparticle-enhancing electrical and thermal transport for carbon nanotube fibers. Appl. Therm. Eng..

[50] Kresse G, Furthmüller J (1996). Efficiency of ab-initio total energy calculations for metals and semiconductors using a plane-wave basis set. Comput. Mater. Sci..

[51] Kresse G, Furthmüller J (1996). Efficient iterative schemes for ab initio total-energy calculations using a plane-wave basis set. Phys. Rev. B Condens. Matter Mater. Phys..

[52] Perdew JP, Burke K, Ernzerhof M (1996). Generalized gradient approximation made simple. Phys. Rev. Lett..

[53] Togo A, Oba F, Tanaka I (2008). First-principles calculations of the ferroelastic transition between rutile-type and CaCl_2_-type SiO_2_ at high pressures. Phys. Rev. B Condens. Matter Mater. Phys..

[54] Li W, Carrete J, Katcho NA, Mingo N (2014). ShengBTE: A solver of the Boltzmann transport equation for phonons. Comput. Phys. Commun..

[55] Madsen GKH, Carrete J, Verstraete MJ (2018). BoltzTraP2, a program for interpolating band structures and calculating semi-classical transport coefficients. Comput. Phys. Commun..

