# Supplementary material for: Thermoelectric transports in pristine and functionalized boron phosphide monolayers
Source: Sci Rep. 2021 May 11;11:10030. doi: 10.1038/s41598-021-89579-5 (PMC8113530; doi:10.1038/s41598-021-89579-5)
Supplement: Supplementary file 1 — Supplementary Information. [file 41598_2021_89579_MOESM1_ESM.pdf]

Supplementary Information

Thermoelectric transports in pristine and functionalized boron phosphide monolayers

Min-Shan Li [1,3], Dong-Chuan Mo [2,3] & Shu-Shen Lyu [2,3,*]

[1] School of Chemical Engineering and Technology, Sun Yat-sen University, Guangzhou, 510275, P. R. China.
[2] School of Materials, Sun Yat-sen University, Guangzhou, 510275, P. R. China.
[3] Guangdong Engineering Technology Research Centre for Advanced Thermal Control Material and System Integration (ATCMSI), Sun Yat-sen University, Guangzhou, 510275, P. R. China.
[*] Correspondence and requests for materials should be addressed to S.-S. L. (email: lvshsh@mail.sysu.edu.cn)

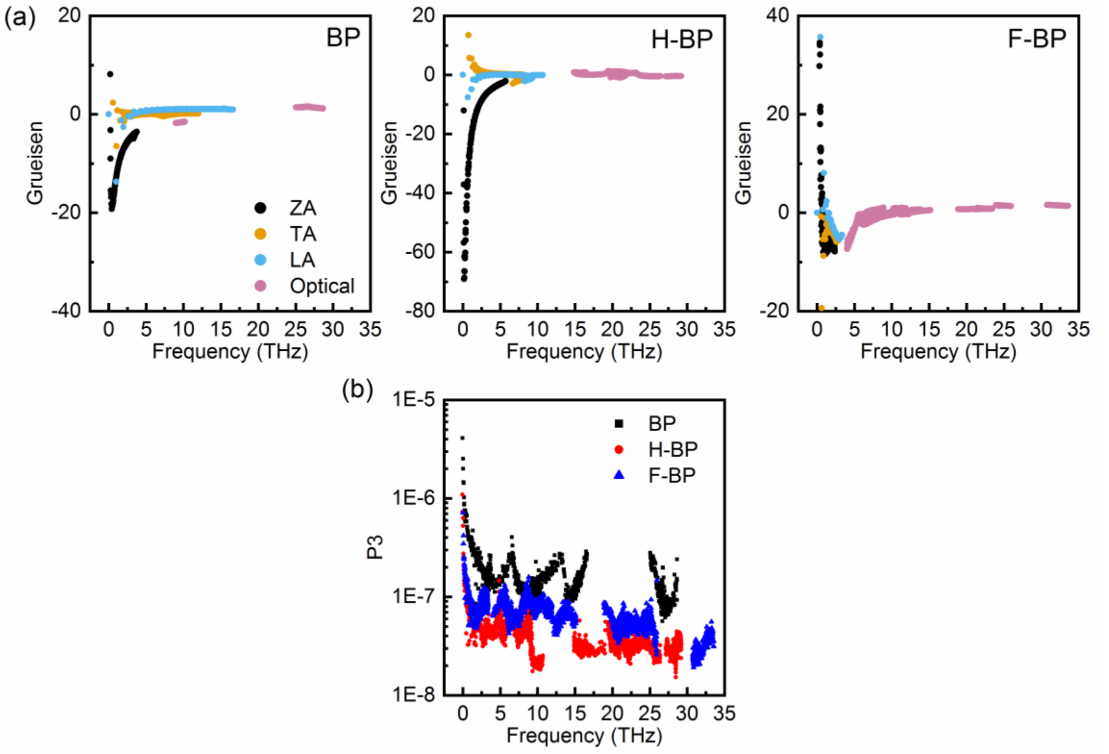

Figure S1. (a) The mode-resolved Grüneisen parameter and
(b) phase space for BP, H-BP and F-BP.

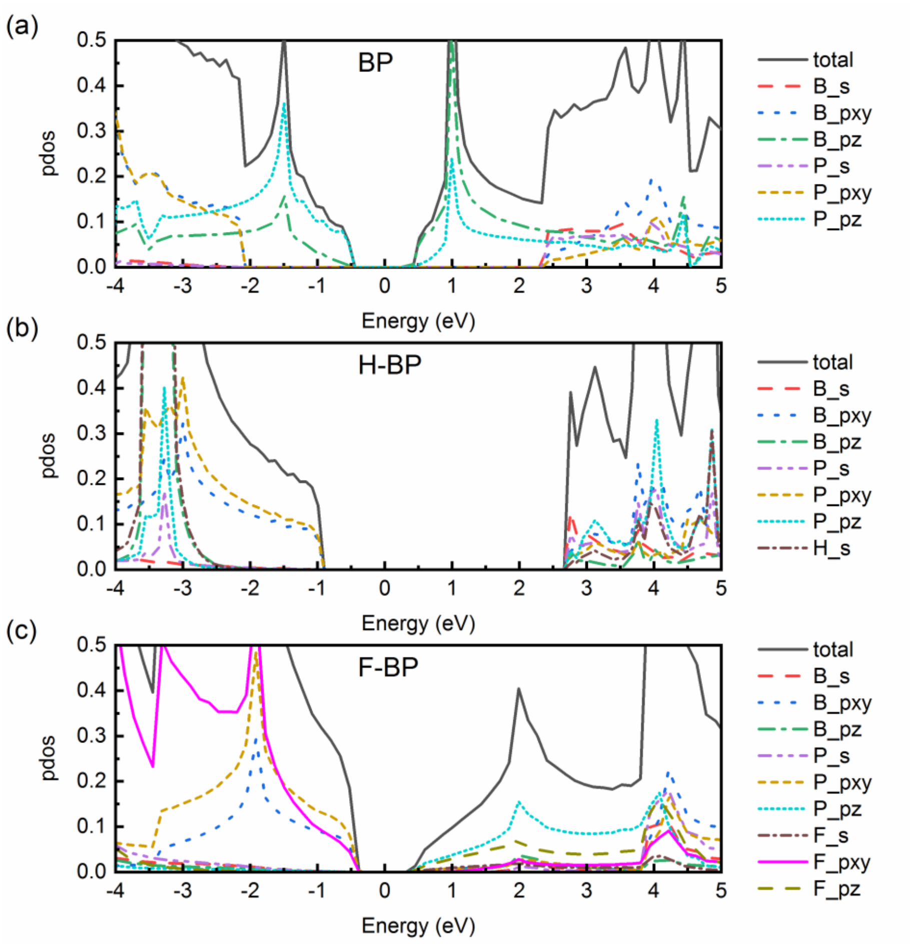

Figure S2. The projected density of states (pDOS) for (a) BP, (b) H-BP and (c) F-BP, respectively.

Table S1. The effective mass m^*, deformation potential constant E_d and carrier relaxation time τ at conduction band maximum (CBM) and valence band minimum (VBM) for BP, H-BP and F-BP.

			m^* (m_e)	E_d (eV)	τ (ps)
BP	CBM	K-G	0.247	2.601	2.424
		K-M	0.291	0.954	
	VBM	K-G	0.249	6.814	0.397
		K-M	0.256	2.489	
H-BP	CBM	M-G	22.242	3.552	6.88×10^{-3}
		M-K	0.156	22.585	
	VBM	G-M	0.897	14.851	3.09×10^{-3}
		G-K	0.879	22.809	
F-BP	CBM	G-M	0.307	2.749	0.175
		G-K	0.327	7.452	
	VBM	G-M	1.173	3.731	0.047
		G-K	1.148	3.570	

m_e is the free electron mass.
